# Visual outcomes of intraocular inflammation after brolucizumab injection in Japanese patients with neovascular age-related macular degeneration

**DOI:** 10.1371/journal.pone.0302295

**Published:** 2024-04-18

**Authors:** Kazushi Hirono, Maiko Maruyama-Inoue, Yasuo Yanagi, Kazuaki Kadonosono

**Affiliations:** Department of Ophthalmology and Micro-technology, Yokohama City University Medical Center, Yokohama, Japan; St. Marianna University School of Medicine, JAPAN

## Abstract

**Purpose:**

This study investigates the visual outcomes of neovascular age-related macular degeneration (nAMD) patients who developed intraocular inflammation (IOI) after intravitreal brolucizumab injection (IVBr).

**Methods:**

We studied 285 eyes of 279 cases diagnosed with nAMD and focused on 18 eyes (6.3%) of 17 cases which developed IOI after IVBr. IVBr was performed either on the initial treatment or for switching of other anti-vascular endothelial growth factor agents during January 2020 to December 2021. We evaluated clinical features and the course of treatment of a 6-month follow-up after IOI occurred.

**Results:**

Of 17 cases, 9 cases were male, 8 cases were female. Baseline logarithm of the minimum angle of resolution(logMAR) best-corrected visual acuity (BCVA) was 0.36, BCVA before IOI occurred was 0.30, and BCVA when IOI occurred was 0.43. 16 eyes (88.9%) had symptoms such as visual loss or floaters when IOI occurred. On the other hand, the remaining 2 eyes (11.1%) had no symptoms. 11 eyes (61.1%) had only IOI, while the remaining 7 eyes (38.9%) had IOI and perivascular sheathing. Steroid sub-tenon injection was performed on 1 eye (5.6%), steroid eye drops were used in 11 eyes (61.1%), and 6 eyes (33.3%) were followed-up without treatment. Neovascular AMD recurred in 16 eyes (88.9%) after IOI occurred and were treated with aflibercept. VA at 3 and 6 months after IOI occurred were significantly improved to 0.34 and 0.30, respectively (*P* = 0.09 at 3 months and *P* = 0.02 at 6 months). The symptoms of patients were improved in all cases. We were able to stop steroid treatment in all cases.

**Conclusions:**

IOI occurred in 6.3% of nAMD patients after IVBr treatment. All of which showed significant improvement from logMAR of 0.43 to 0.30 with steroid treatment or without any treatment. We should consider the possibility of IOI after IVBr as a complication, however, they have a relatively good prognosis if treated at an early stage.

## Introduction

Aging population is gradually increasing, resulting in more patients with age-related macular degeneration (AMD). It is now widely known to treat neovascular AMD (nAMD) with anti-vascular endothelial growth factor (VEGF) intravitreal injections such as ranibizumab (Lucentis, Genentech, Inc., South San Francisco, CA) and aflibercept (Eylea, Regeneron Pharmaceuticals, Tarrytown, NY, USA) [1, 2].

Recently, brolucizumab (Beovu, Novartis International, Basel, Switzerland), an approximate 26-kDa single-chain antibody fragment, was introduced as a new anti-VEGF agent along with reports of good progress in the course of treatment [3]. Our group showed that brolucizumab has better efficacy and could decrease injection frequency for patients compared to aflibercept after switching to brolucizumab [4, 5]. However, there have been reports of intraocular inflammation (IOI) in patients treated with brolucizumab [6, 7]. IOI may result in sight-threatening outcomes if not treated properly. Treatment regimens have also been reported for IOI, retinal vasculitis, and retinal vascular occlusion events in patients treated with brolucizumab.

Based on inflammation severity, it is recommended to treat IOI with potent topical corticosteroids, and additional intravitreal or systemic corticosteroids if needed [8–12]. Retinal vasculitis is recommended to be treated with either potent topical, intravitreal, and/or systemic corticosteroids, supplemented with implant and/or sub-tenon corticosteroids [8–12]. Potent corticosteroid treatment (systemic, intravitreal, or topical) is also recommended in patients with retinal vascular occlusive events [8–12]. Vitrectomy or panretinal photocoagulation should be considered if there are severe occlusive events [13]. However, visual outcomes after IOI occurred is still uncertain. This study evaluated clinical features and the course of treatment of a 6-month follow-up after IOI occurred.

## Patients and methods

We retrospectively studied 285 eyes of 279 patients diagnosed with nAMD who were followed for more than 6 months and focused on 18 eyes (6.3%) of 17 cases which developed IOI after intravitreal brolucizumab injections (IVBr) and at Yokohama City University Medical Center. IVBr was performed either on the initial treatment or for switching of other anti-VEGF agents during the period of January 2020 to December 2021. Sixty-nine out of 279 eyes (24.7%) treated with IVBr were diagnosed as polypoidal choroidal vasculopathy (PCV) and remaining 210 eyes (75.3%) were non-PCV. In patients with treatment-naïve AMD, the patients received 3 monthly IVBr in the loading phase. In the maintenance phase, patients were treated with IVBr every 12 weeks unless any fluid or new hemorrhage were identified. If fluid or new hemorrhage were seen, the interval of IVBr was shortened to every 8 weeks. In patients with AMD who switched to brolucizumab, they were treated by the same interval as the last interval of previous anti-VEGF agents. The shortest intervals was 4 weeks and the longest intervals was 12 weeks when being switched. Then, treatment intervals were adjusted for 1–2 weeks, based on the intervals at which the disease recurs. The ethics committee of Yokohama City University Medical Center approved the study, which was conducted according to the principles of the Declaration of Helsinki. All patients provided written informed consent before their medical record data were used in this research. The data were accessed for research purposes in February 1^st^, 2022. The authors had access to information that could identify individual participants during or after data collection.

Demographic information including age, gender, past medical history, smoking, lens status, intraocular surgery within a year before IVBr, AMD subtype (PCV or non-PCV), treatment naïve or switching was obtained for each patient.

IOI was defined when patients had clinical findings such as inflammation of the anterior chamber including iritis and keratic precipitates, vitreous opacity, or perivascular sheathing with symptoms such as floaters, blurred or decreased vision. Patients who developed IOI stopped IVBr and were followed-up without treatment or treated with either topical and/or sub-tenon corticosteroids. Patients were followed-up every month after IOI occurred for 6 months. During the follow-up period, patients switched to other anti-VEGF agents if AMD recurred. All eyes except those that developed IOI continued to use brolucizumab.

The primary outcome measure was the changes of logarithm of the minimum angle of resolution (logMAR) best-corrected visual acuity (BCVA) before and after IOI. LogMAR BCVA before IOI occurred was defined as baseline. Secondary outcome measures were patient’s information of whether retinal vasculitis or retinal vascular occlusion occurred, days from first IVBr to IOI, days from the last IVBr to IOI, and the total number of IVBr before IOI.

The Wilcoxon signed rank test was conducted to compare the baseline parameters with those at each time point. Fisher’s exact test was used to compare the percentage of IOI between PCV and non-PCV group. Statistical analyses were performed with the statistical programming language R (ver. 4.2.2; The R Foundation for Statistical Computing). *P*-values < 0.05 were considered statistically significant.

## Results

### Patient characteristics

**Fig 1:**
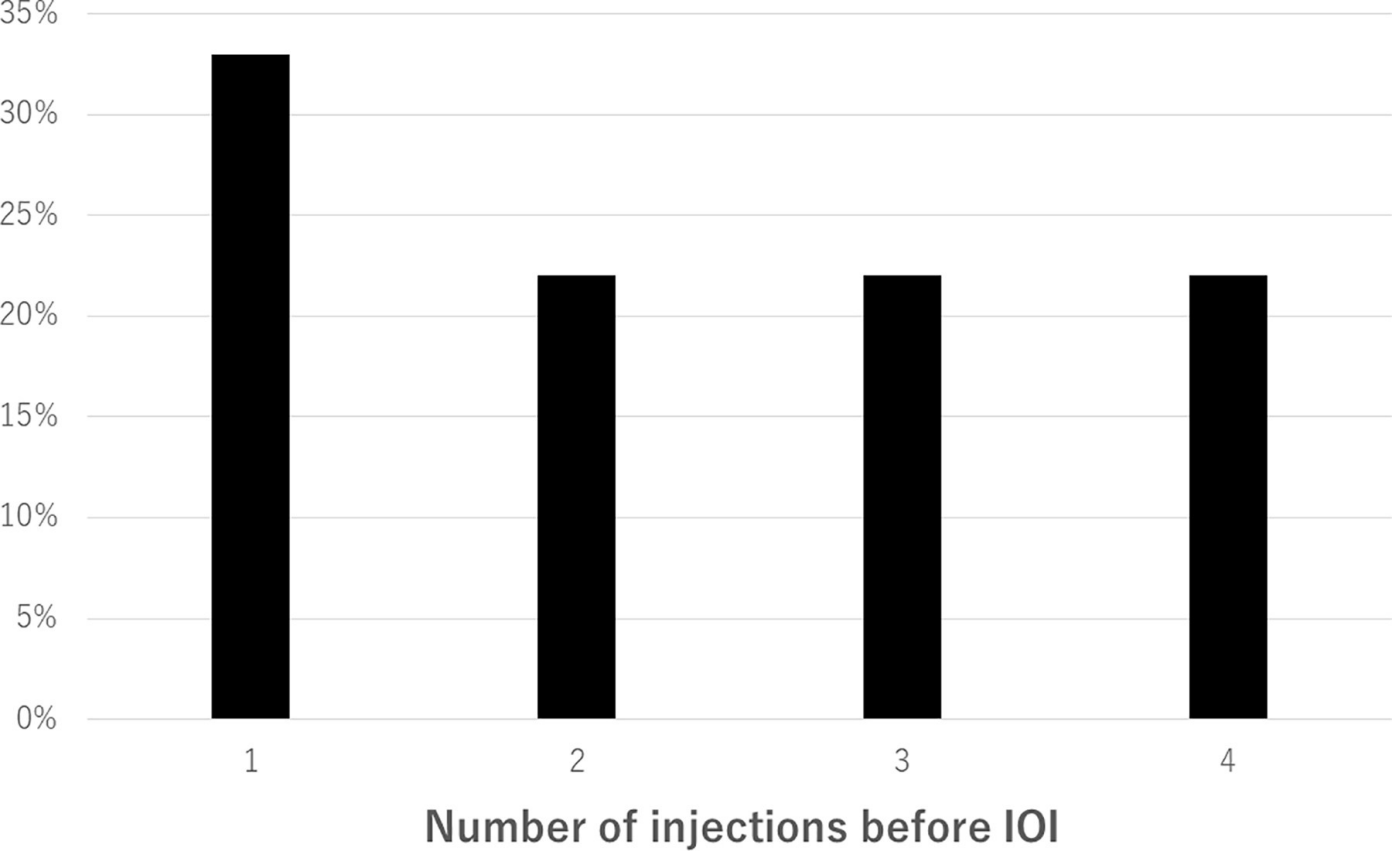
Number of IVBr before IOI occurred. IOI occurred at a higher percentage after the first injections, compared to two or more injections.

Patient baseline characteristics and clinical data are shown in Table 1. Eighteen eyes (6.3%) in 17 patients (9 male, 8 female) which developed IOI after IVBr were assessed at the 6 months follow-up evaluation. The patients’ age ranged from 58 to 95 years old (mean age, 76.0 ± 11.2 years). 12 eyes (66.7%) were treatment naïve and the remaining 6 eyes (33.3%) were switched from other anti-VEGF agents. Eight out of 18 eyes (44.4%) had PCV. Therefore, 8 of 69 eyes (11.6%) in patients with PCV and 10 of 210 (4.8%) in patients with non-PCV developed IOI, which was no significant difference between two groups (*P* = 0.09). No eyes had received intraocular surgery within 1 year. Three, eight, and zero out of 17 patients (17.6%, 47.1%, and 0%) had medical history of diabetes mellitus, hypertension, and rheumatological disease, respectively. Six out of 17 patients (35.3%) were current or past smokers. The average number of brolucizumab injections before IOI occurred ranged from 1 to 4 times (mean 2.33 ± 1.15) (Fig 1). The number of days from the first brolucizumab injection from when IOI occurred ranged from 14 to 245 days (mean 102.7 ± 71.8 days). The number of days from the last brolucizumab injection from when IOI occurred ranged from 14 to 98 days (mean 47.8 ± 22.0 days).

**Table 1:** Baseline characteristics and clinical data of patients who developed IOI after IVBr.

Case	Patients	R/L	Age	Symptom	Gender	Lens status	PCV/nonPCV	Naïve or switching	History of surgery within a year	Vasculitis	Number of IVBr before IOI	Number of days since first IVBr until IOI	Number of days after last IVBr until IOI	DM	HT	RD	Smoking	VA when IOI occured	VA 6 months after IOI occured	Treatment
1	1	R	64	Floaters	M	P	PCV	S	-	-	1	37	37	+	+	-	+	20/16	20/16	None
2	2	R	58	Floaters	M	P	PCV	N	-	-	2	52	14	-	-	-	+	20/22	20/25	Topical
3	3	L	72	Floaters	M	I	PCV	N	-	-	3	126	63	-	+	-	-	20/28	20/25	Topical
4	4	L	81	Floaters	F	P	nonPCV	N	-	-	4	169	56	-	-	-	+	20/250	20/250	Topical
5	5	R	81	Floaters	M	I	nonPCV	N	-	-	1	35	35	-	-	-	+	20/50	20/50	Topical STTA
6	6	R	82	Floaters	F	P	nonPCV	N	-	-	3	104	47	-	+	-	-	20/25	20/25	Topical
7	7	L	91	Floaters	F	P	nonPCV	N	-	-	4	220	70	-	-	-	-	20/25	20/33	None
8	8	L	58	Visual loss	F	I	nonPCV	N	-	+	2	126	42	+	-	-	-	20/40	20/33	Topical
9	9	L	95	None	F	P	nonPCV	S	-	+	4	231	63	+	+	-	-	20/66	20/66	None
10	10	R	80	Blurred vision	M	P	PCV	S	-	+	1	49	49	-	-	-	+	20/285	20/200	Topical
11	11	L	63	None	M	I	nonPCV	N	-	+	4	245	98	-	-	-	-	20/16	20/20	None
12	12	R	90	None	F	P	nonPCV	N	-	+	3	140	84	-	+	-	+	20/200	20/133	None
13	12	L	90	None	F	P	nonPCV	N	-	+	3	112	56	-	+	-	+	20/25	20/28	None
14	13	R	72	Floaters	F	I	PCV	N	-	+	2	49	21	-	+	-	-	20/50	20/28	Topical
15	14	L	85	Floaters	M	I	PCV	S	-	-	2	70	42	-	+	-	-	20/28	20/20	Topical
16	15	L	72	Blurred vision	M	I	nonPCV	N	-	-	1	28	28	-	-	-	-	20/200	20/25	Topical
17	16	R	71	Eye pain	F	I	PCV	S	-	-	1	14	14	-	-	-	-	20/200	20/66	Topical
18	17	R	67	Floaters	M	I	nonPCV	S	-	-	1	42	42	-	+	-	-	20/50	20/25	Topical

PCV = polypoidal choroidal vasculopathy; IVBr = intravitreal brolucizumab injection; IOI = intraocular inflammation; DM = diabetes mellitus; HT = hypertension; RD = rheumatological disease; VA = visual acuity; STTA = subtenon’s triamcinolone acetonide injection; R = right eye; L = left eye; M = male; F = female; P = phakic; I = intraocular lens S = switching; N = naive

### Visual acuity outcomes

**Fig 2:**
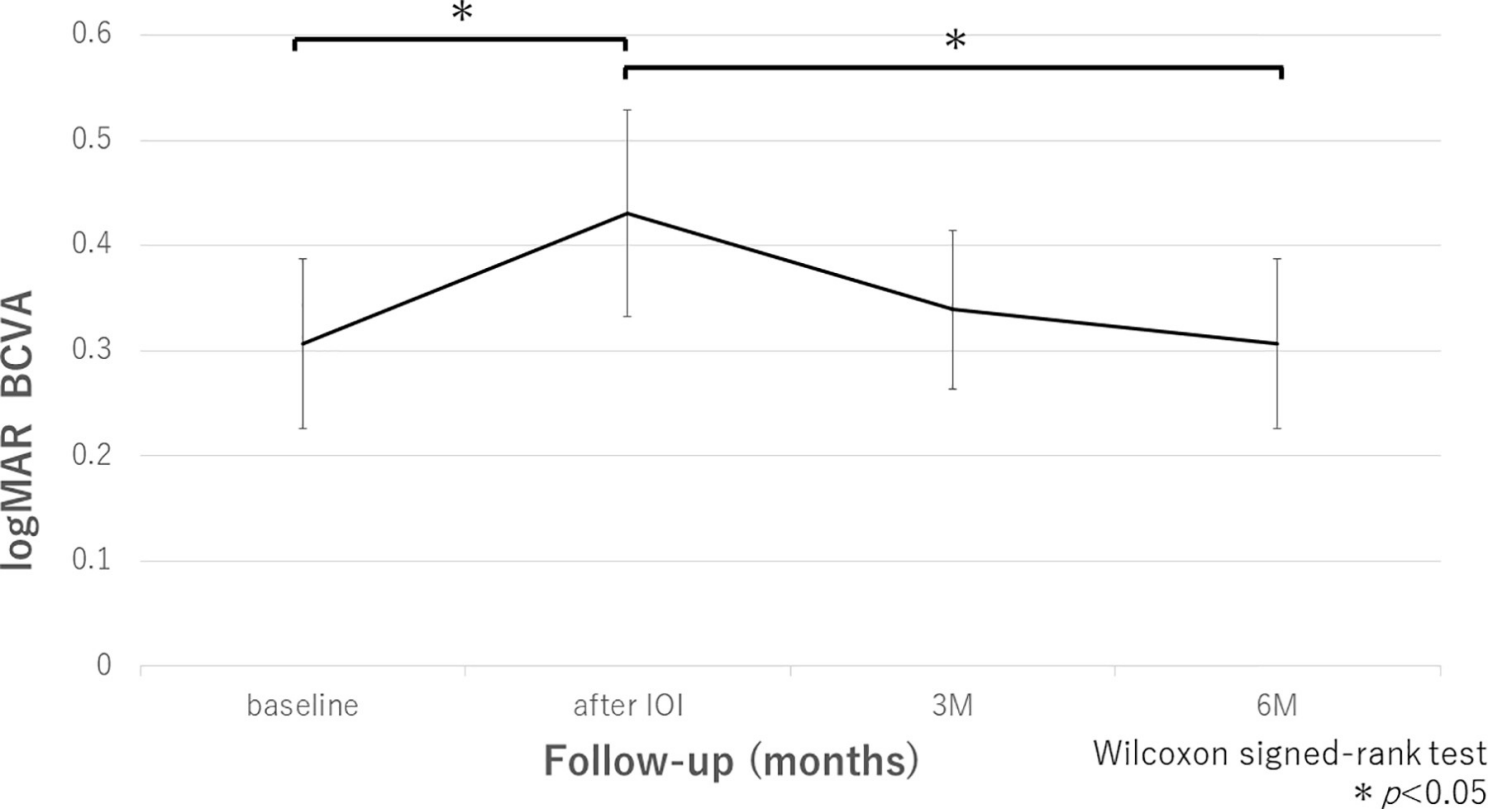
Changes in the mean logMAR BCVA ± standard error from baseline to 6 months after IOI occurred. The mean logMAR BCVA when IOI occurred decreased significantly compared with before IOI occurred (*P* = 0.02). The mean logMAR BCVA improved significantly after 6 months compared with after IOI occurred (*P* = 0.02).

The mean baseline logMAR BCVA, which was the last visit before IOI occurred, was 0.30 ± 0.34. The mean logMAR BCVA when IOI occurred was 0.43 ± 0.41, which decreased significantly compared with BCVA when IOI occurred (*P* = 0.02) (Fig 2). The mean logMAR BCVA at 3 and 6 months after IOI occurred were 0.34 ± 0.32 and 0.30 ± 0.34, respectively. BCVA at 6 months, not at 3 months, significantly improved compared with the baseline BCVA (*P* = 0.09 and *P* = 0.02 at 3 and 6 months, respectively) (Fig 2).

### Symptoms

**Fig 3:**
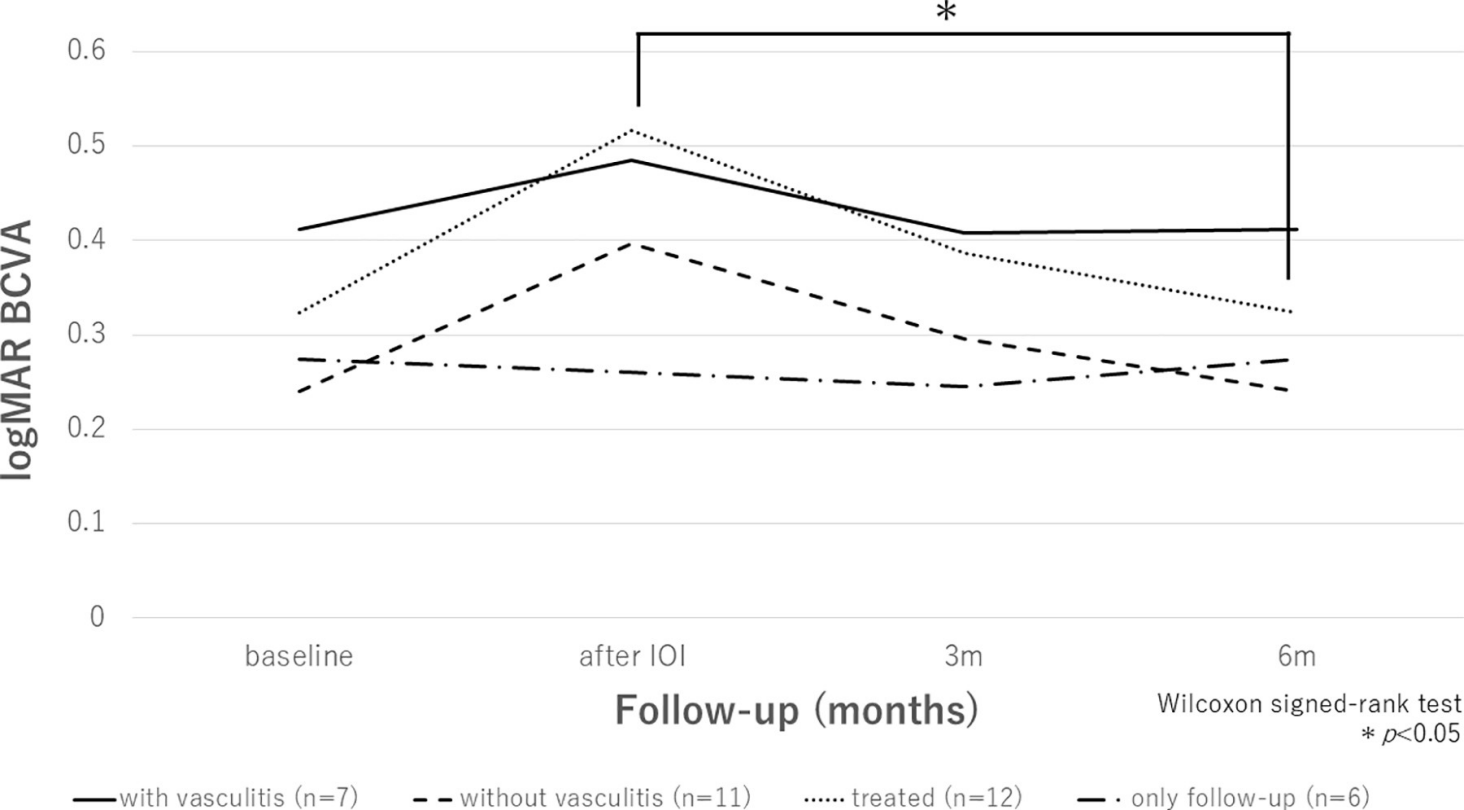
Comparison of changes in the mean logMAR BCVA in different groups from baseline to 6 months after IOI occurred. The different groups include eyes with vasculitis, without vasculitis, treated, and untreated. Only treated group showed significant improvement of logMAR BCVA at six months after IOI compared to after IOI (*P* = 0.15, *P* = 0.10, *P* = 0.01, and *P* = 0.87 in eyes with vasculitis, without, treated, and untreated groups, respectively).

16 out of 18 eyes (88.9%) had symptoms and clinical findings such as inflammation of the anterior chamber including iritis and keratic precipitates, floaters, ocular discomfort, blurred or decreased vision, and vitreous opacity when IOI occurred while the remaining two eyes (11.1%) had none. Seven out of 18 eyes (38.9%) developed retinal vasculitis and perivascular sheathing. No eyes developed retinal vascular occlusion. There were also no patients who showed increased intraocular pressure after the IOI occurred. The changes in logMAR BCVA in eyes with vasculitis, without vasculitis, treated, and untreated are shown in Fig 3. Only treated group showed significant improvement of logMAR BCVA at six months after IOI compared to after IOI (*P* = 0.15, *P* = 0.10, *P* = 0.01, and *P* = 0.87 in eyes with vasculitis, without, treated, and untreated groups, respectively).

### Treatment

After the development of IOI, patients were followed up 1 to 2 weeks after IOI occurred, followed by one month interval visits. We prescribed corticosteroids in patients who had some symptoms such as moderate to severe floaters, vitreous opacity, and anterior chamber inflammation. 11 out of 18 eyes (61.1%) were treated with only topical corticosteroids. 1 out of 18 eyes (5.6%) were treated with topical corticosteroids (betamethasone eyedrops) with additional sub-tenon corticosteroid injections.

Six out of 18 eyes (33.3%) were followed-up without any treatment. Two of 6 patient had mild floaters. Symptoms of these patients improved monotonously without affecting visual acuity. The other 4 patients had no symptoms and perivascular sheathing alone. All patients still have perivascular sheathing in the same area but have no change in symptoms or visual acuity at 6 months follow-up.

**Fig 4:**
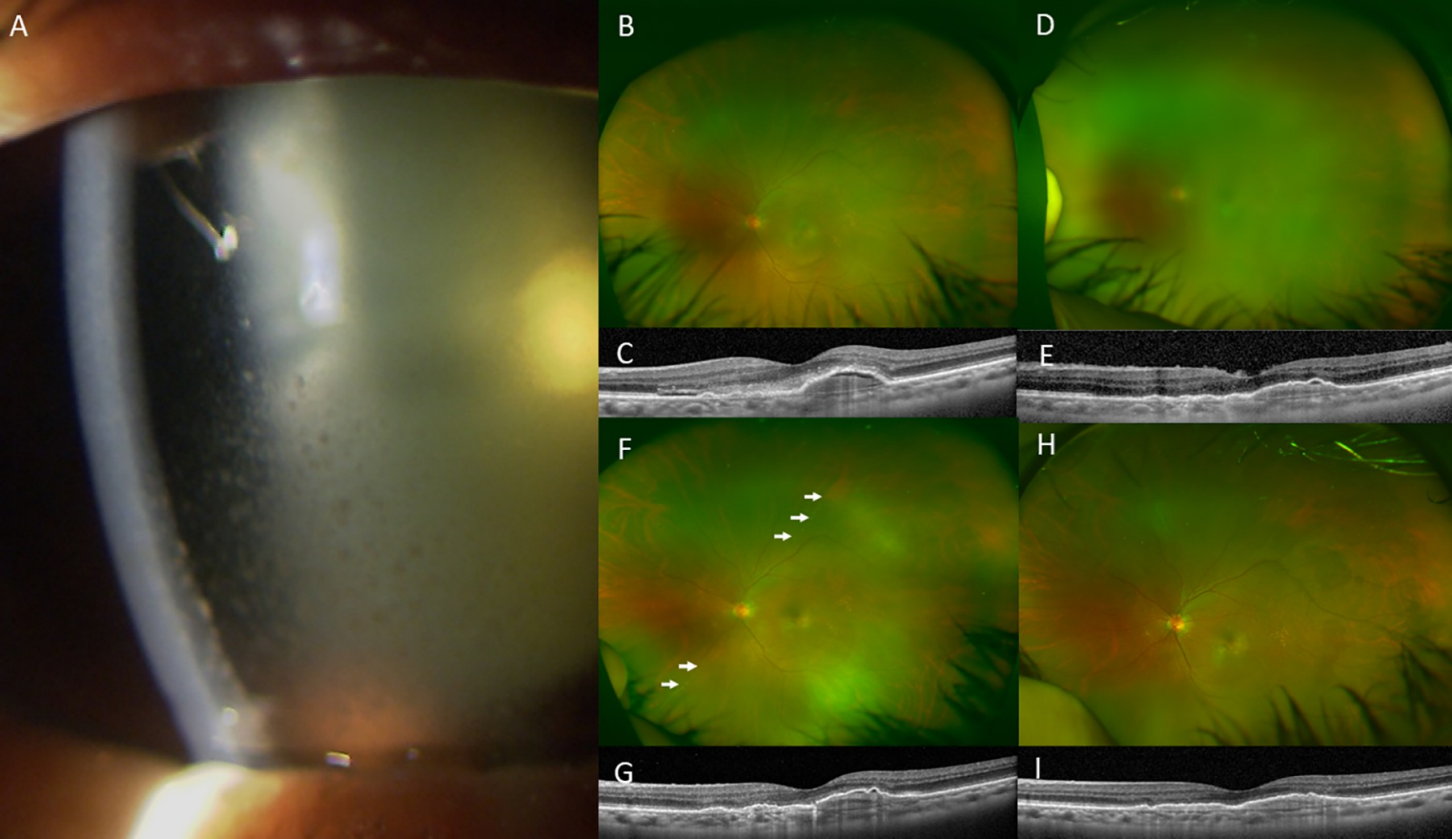
Images from case 16 with IOI after IVBr. A, Photo of the anterior segment showing keratic precipitates and anterior chamber cells. B, Fundus photograph obtained at baseline. C, OCT image at baseline showing pigment epithelial detachment (PED), small subretinal fluid (SRF) with fibrin. D, Fundus photograph obtained after IOI occurred 4 weeks after the first IVBr showing vitreous opacity reducing clarity of the image. IOI was followed-up with topical steroid. E, OCT image after IOI showing reduced PED, resolution of SRF and fibrin, and vitreous cells along with reduced clarity of the image. F, Fundus photograph obtained 3 months after IOI occurred showing less vitreous opacity with increased clarity of the image. Retinal vasculitis was also seen with vessel sheathing (white arrows). G, OCT image obtained 3 months after IOI occurred showing increased clarity of the image. H, Fundus photograph obtained 6 months after IOI occurred with increased clarity with no vitreous opacity. Retinal vasculitis and sheathing of vessels were also resolved. I, OCT image obtained 6 months after IOI showing further reduced PED and clarity maintained.

**Fig 5:**
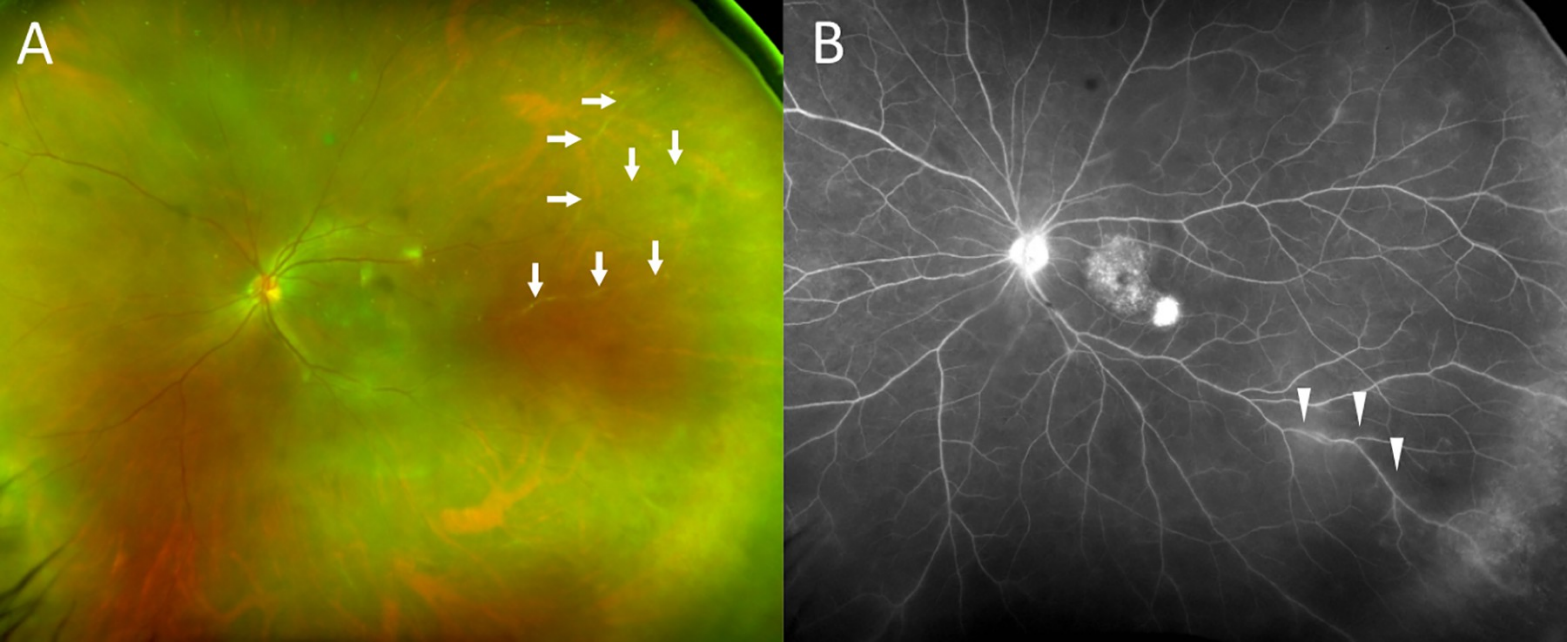
Images from case 8 with IOI after IVBr. A, Fundus photograph showing perivascular sheathing and vasculitis (white arrows). B, Fundus fluorescein angiography photograph showing vascular leakage (white arrowheads).

Sixteen out of 18 eyes (88.9%) recurred AMD during the 6 months follow-up. Two eyes switched to ranibizumab, 1 eye switched to faricimab (Vabysmo, Roche/Genentech, Basel, Switzerland), and 13 eyes switched to aflibercept. At 6 months after IOI, the symptoms of patients were improved in all cases. We were able to stop treatment for IOI in all cases. Representative cases are shown in Figs 4 and 5.

## Discussion

Although cases of IOI development after IVBr have been reported, there are few reports of a treatment outcome. In the current study, we found that visual acuity and symptoms of these patients can be significantly improved if IOI is found early and treated appropriately.

IOI could occur in all anti-VEGF drugs. The incidence of IOI in aflibercept-related IOI was 2.25% in a previous study [14]. Another study showed the incidence of bevacizumab-related IOI was approximately 0.3% [15]. According to the TENAYA and LUCERNE data, the incidence of IOI in faricimab-treated patients was 1.6% in the Asian country subgroup, and 2.0% in the non-Asian country subgroup [16]. However, the main cause and mechanism of IOI and vascular occlusive events is still uncertain. Suggested possible hypotheses for the mechanism of these events include formation of local anti-bodies to the drug itself, other protein byproducts, differences in pH, immune status, and causative comorbidities [8, 14, 15, 17–20]. It is also reported that IOI risk factors include female sex, a history of retinal vascular occlusion, and bilateral brolucizumab treatment on the same day [21–23].

Additionally, a differential diagnosis of chronic endophthalmitis should be made when IOI occurs. However, it is known that IOI could happen from the vitreous as a origin and anterior inflammation is relatively mild [7]. On the other hand, endophthalmitis tends to show severe anterior chamber inflammation such as hypopyon, fibrin [24]. Therefore, it is not difficult to distinguish them clinically. However, it might be better to submit anterior chamber culture if it is difficult to make a decision.

According to the SRC review of HAWK and HARRIER data, approximately 4.6% of eyes developed IOI after IVBr, which was similar to our results [3]. Matsumoto et al. described that 22.1% of eyes developed IOI after IVBr [25]. Another report presented that 9.4% of eyes developed IOI after IVBr [20, 24]. It is also reported that patients developed IOI with varying severities including retinal vasculitis and retinal vascular occlusion, typically within 2 months of their last IVBr [7–12]. In our study, the mean number of days from the last IVBr to when IOI occurred was 47.83 days showing similar results. However, some patients were diagnosed as IOI in 63–98 days after the last IVBr. Because they had no symptoms, we found IOI at the scheduled visit. Our current study also showed that the mean number of days from the first IVBr to when IOI occurred was 102.72 days. Although it is important to be cautious after the first IVBr, we should pay attention even after the loading phase because IOI could occur.

Previous reports recommended that we should perform fluorescein angiography (FA) and indocyanine green angiography (ICGA) on patients to confirm retinal vasculitis or retinal vascular occlusion after IVBr [16, 25, 26]. In this study, 6 eyes developed retinal vasculitis with perivascular sheathing, and no eyes developed retinal vascular occlusion. However, we haven’t routinely performed either FA nor ICGA. It doesn’t mean that we recommend dismissing to perform FA or ICGA. These angiographies should be done if development of severe retinal vasculitis or retinal vein occlusion is strongly suspected to confirm ischemic areas [25].

The current results showed that the mean logMAR BCVA changed from 0.43 when IOI occurred to 0.30 at 6 months indicating significant improvement. This indicates that BCVA can be improved if IOI is found early and treated properly from the point of development. Based on inflammation severity, IOI is recommended to be treated with potent topical corticosteroids with additional intravitreal corticosteroids and systemic corticosteroids if necessary [7–12]. In the current study, only 1 eye (5.5%) with IOI was treated with sub-tenon corticosteroids, and 11 eyes were treated with only topical corticosteroids. The remaining 6 eyes were followed-up without treatment. Of the 6 eyes which were observed without treatment, 2 eyes had just IOI, 4 eyes had retinal vasculitis with perivascular sheathing. These four eyes with retinal vasculitis and perivascular sheathing, had no symptoms and were found incidentally at the planned visit. Since these patients had no decrease of visual acuity, and the fact that it can be predicted that time has passed to some extent after IOI actually occurred, physicians decided to follow-up without any treatment. As a result, all cases showed improvement of subjective symptoms and visual acuity during the follow-up. Although symptoms and severity varied in these cases, it can be said that not all cases require corticosteroid treatment for improvement of BCVA. On the other hand, additional sub-tenon corticosteroids and systemic corticosteroids, as being recommended in the previous reports, might accelerate the amelioration of IOI and prevent the development of retinal vascular occlusion compared to no treatment [7–12]. Although an optimal treatment protocol for IOI should be considered in the future, we should select appropriate treatment by evaluating the severity of IOI in each patient.

The current study had several limitations, the most important being its retrospective design and small number of patients. Furthermore, since all patients were Japanese, the results of this study cannot be generalized for other racial or ethnic groups. Also, there are differences in treatment standards in each patient due to several different physicians participating in this study. Since there were no patients with retinal vascular occlusions in our study, we didn’t describe the efficacy of corticosteroid on retinal vascular occlusions. Furthermore, some patients were diagnosed as IOI at a scheduled visit, which was more than 2 months after last injection. These cases might be difficult to differentiate IOI from uveitis. More cases and further investigations are needed to establish appropriate diagnosis and management for IOI after IVBr.

In conclusion, improvement of subjective symptoms and visual function were seen in cases that developed IOI after IVBr during a 6-month period, most of which was treated with either topical corticosteroid treatment or none. The current results suggest that singular usage of topical corticosteroids might be potential treatment option for IOI after IVBr unless retinal vascular occlusion developed.

## References

[1] Rosenfeld PJ, Brown DM, Heier JS, et al. Ranibizumab for neovascular age-related macular degeneration. N Engl J Med. 2006;355:1419–1431 doi: 10.1056/NEJMoa054481 17021318

[2] Heier JS, Brown DM, Chong V, Korobelnik JF, et al. Intravitreal aflibercept(VEGF trap-eye) in wet age-related macular degeneration. Ophthalmology. 2012;119:2537–2548. doi: 10.1016/j.ophtha.2012.09.006 23084240

[3] Mones J, Srivastava SK, Jaffe GJ, Tadayoni R, Albini TA, Kaiser PK, et al. Risk of inflammation, retinal vasculitis, and retinal occlusion-related events with brolucizumab: post hoc review of HAWK and HARRIER. Ophthalmology. 2021;128:1050–1059. doi: 10.1016/j.ophtha.2020.11.011 33207259

[4] Kitajima Y, Maruyama-Inoue M, Ikeda S, Ito A, et al. Short-term outcomes of switching to brolucizumab in Japanese patients with neovascular age-related macular degeneration. Jpn J Ophthalmol. 2022;66:511–517.0 doi: 10.1007/s10384-022-00940-1 36149566

[5] Ito A, Maruyama-Inoue M, Kitajima Y, et al. One-year outcomes of intravitreal brolucizumab injections in patients with polypoidal choroidal vasculopathy. Scientific Rep. 2022;12:7987. doi: 10.1038/s41598-022-12216-2 35568780 PMC9107469

[6] Agostini H, Mulyukov Z, Tsilimbaris M, et al. Comparison of the efficacy of brolucizumab with natural disease progression in wet AMD using clinical data from the phase III HAWK and HARRIER trials and modelled placebo data. Curr Eye Res. 2020;45:1298–1301. doi: 10.1080/02713683.2020.1731832 32065533

[7] Baumal CR, Spaide RF, Vajzovic L, et al. Retinal vasculitis and intraocular inflammation after intravitreal injection of brolucizumab. Ophthalmology. 2020;127:1345–1359. doi: 10.1016/j.ophtha.2020.04.017 32344075

[8] Baumal CR, Bodaghi B, Singer M, et al. Expert opinion on management of intraocular inflammation, retinal vasculitis, and vascular occlusion after brolucizumab treatment. Ophthalmol Retina. 2021;5:519–527. doi: 10.1016/j.oret.2020.09.020 33007521

[9] Haug SJ, Hien DL, Uludag G, et al. Retinal arterial occlusive vasculitis following intravitreal brolucizumab administration. Am J Ophthalmol Case Rep. 2020;18:100680. doi: 10.1016/j.ajoc.2020.100680 32258827 PMC7125319

[10] Jain A, Chea S, Matsumiya W, et al. Severe vision loss secondary to retinal arteriolar occlusions after multiple intravitreal brolucizumab administrations. Am J Ophthalmol Case Rep. 2020;18:100687. doi: 10.1016/j.ajoc.2020.100687 32280811 PMC7139151

[11] Witkin A, Hahn P, Murray T, et al. Occlusive retinal vasculitis following intravitreal brolucizumab. J Vitreoretin Dis. 2020, 247412642093086. doi: 10.1177/2474126420930863 32789284 PMC7418897

[12] Kondapalli SSA. Retinal vasculitis after administration of brolucizumab resulting in severe loss of visual acuity. JAMA Ophthalmol. 2020 Aug 6. doi: 10.1001/jamaophthalmol.2020.2810 32761135

[13] Flaxel CJ, Adelman RA, Bailey ST, et al. Retinal and Ophthalmic Artery Occlusions Preferred Practice Pattern (R). Ophthalmology. 2020;127:p259–287. 31757501 10.1016/j.ophtha.2019.09.028

[14] Goldberg RA, Shah CP, Wiegand TW, et al. Noninfectious inflammation after intravitreal injection of aflibercept: clinical characteristics and visual outcomes. Am J Ophthalmol. 2014;158:733–737. doi: 10.1016/j.ajo.2014.06.019 24983791

[15] Georgopoulos M, Polak K, Prager F, et al. Characteristics of severe intraocular inflammation following intravitreal injection of bevacizumab (Avastin). Br J Ophthalmol. 2009;93:457–462. doi: 10.1136/bjo.2008.138479 19033289

[16] Hashimoto Y, Inoda S, Takahashi H, et al. Factors associated with intraocular inflammation in neovascular age-related macular Degeneration patients treated with brolucizumab. Invest Ophthalmol Vis Sci. 2024;65(1):8 doi: 10.1167/iovs.65.1.8 38170536 PMC10768698

[17] Enriquez AB, Baumal CR, Crane AM, et al. Early experience with brolucizumab treatment of neovascular age-related macular degeneration. JAMA Ophthalmol. 2021;139:441–448. doi: 10.1001/jamaophthalmol.2020.7085 33630045 PMC7907988

[18] Khanani AM, Zarbin MA, Barakat MR, et al. Safety outcomes of brolucizumab in neovascular age-related macular degeneration: results from IRIS registry and komodo healthcare map. JAMA Ophthalmol. 2022;140:20–28. 34817566 10.1001/jamaophthalmol.2021.4585 PMC8613703

[19] Mukai R, Matsumoto H, Akiyama H. Risk factors for emerging intraocular inflammation after intravitreal brolucizumab injection for age-related macular degeneration. PLoS One. 2021;16. doi: 10.1371/journal.pone.0259879 34871313 PMC8648104

[20] Wykoff CC, Matsumoto H, Barakat MR, et al. Retinal vasculitis or vascular occlusion after brolucizumab for neovascular age-related macular degeneration: a systematic review of real-world evidence. Retina. 2023;43(7):1051–1063 doi: 10.1097/IAE.0000000000003769 36893438 PMC10278563

[21] Sadda SR, Holz FG, Staurenghi G, et al. The importance of imaging to identify early signs of intraocular inflammation expert opinion for brolucizumab. Ophthalmologica. 2022;245:588–591. doi: 10.1159/000526703 36130526 PMC9843731

[22] Tranos P, Karasavvidou EM, Gkorou O, et al. Optical coherence tomography angiography in uveitis. J Ophthalmic Inflamm Infect. 2019;9:21. doi: 10.1186/s12348-019-0190-y 31873858 PMC6928173

[23] Herbort CP. Fluorescein and indocyanine green angiography for uveitis. Middle East Afr J Ophthalmol. 2009;16:168–187. doi: 10.4103/0974-9233.58419 20404985 PMC2855659

[24] Maruko I, Okada AA, Iida T, et al. Japan AMD Research Consortium. Brolucizumab-related intraocular inflammation in Japanese patients with age-related macular degeneration: a short-term multicenter study. Graefes Arch Clin Exp Ophthalmol. 2021;259(9):2857–2859 33723637 10.1007/s00417-021-05136-w PMC8380561

[25] Matsumoto H, Hoshino J, Mukai R, et al. One-year results of treat-and-extend regimen with intravitreal brolucizumab for treatment-naïve neovascular age-related macular degeneration with type 1 macular neovascularization. Sci Rep. 2022;12(1):8195 35581196 10.1038/s41598-022-10578-1 PMC9114020

[26] Anderson WJ, da Cruz NFS, Lima LH, Emerson GG, et al. Mechanisms of sterile inflammation after intravitreal injection of antiangiogenic drugs: a narrative review. Int J Retina Vitreous. 2021;7(1):37 doi: 10.1186/s40942-021-00307-7 33962696 PMC8103589

